# Strategies to minimize inequity in COVID-19 vaccine access in the US: Implications for future vaccine rollouts

**DOI:** 10.1016/j.lana.2021.100138

**Published:** 2021-12-08

**Authors:** Anindit Chhibber, Aditi Kharat, Khanh Duong, Richard E. Nelson, Matthew H. Samore, Fernando A. Wilson, Nathorn Chaiyakunapruk

**Affiliations:** a College of Pharmacy, University of Utah, Salt Lake City, UT, USA; b Division of Epidemiology, University of Utah School of Medicine, Salt Lake City, UT, USA; c IDEAS Center, Veterans Affairs Salt Lake City Healthcare System, Salt Lake City, UT, USA; d Matheson Center for Health Care Studies, University of Utah, Salt Lake City, UT, USA

Despite the successful development of vaccines and one of the fastest vaccination program implementations in the world, equitable access to COVID-19 vaccines remains a huge challenge in US.^1^ As of November 1, 2021, data across the US^2^ demonstrates that Black and Hispanic populations are being vaccinated at a lower rate compared to their white counterparts. Across 43 states, Black and Hispanic people are being vaccinated at 7% and 2% lower rates, respectively compared to the white population.^2^ In some states, such as Connecticut, Black and Hispanic people are being vaccinated at 15 and 8% lower rates compared to the white population.^2^ Although there is variability across states in vaccination levels and the inequity gap has been closing over time^2^, inequity still poses a barrier to society wide attainment of ‘fair and just’ health.

COVID-19 vaccine rollout strategies during 2021 have varied substantially across states and little^3^ is known regarding the role of disparities beyond racial equity played in these different strategies. A question of interest is whether state-level vaccine rollout plans considered comprehensive equity and whether compiling a list of vaccine rollout strategies to minimize inequity in vaccine access for future vaccine rollout plans can be beneficial. From an analysis^4^ calculating diversity across six different categories: socioeconomic, cultural, economic, household, religious and political diversity for all US states, we selected 5 of the most diverse US states (California, Texas, Hawaii, New Jersey and New York) and 5 of the least diverse (Montana, New Hampshire, Vermont, Maine and West Virginia). The rollout plans of these states were then analyzed to assess the consideration of comprehensive equity.

Overall, the vaccine rollout strategies that we identified included components related to equity. The most common equity-related components found in these plans were place of residence, race/ethnicity, occupation, education and socioeconomic status; while gender/sex, religion and social capital often missing. The state-level vaccine rollout strategies can be summarized into five domains: prioritization, communication, access, safety and trust, and monitoring (Table 1). Rollout plans from more diverse states like California and Hawaii included more inequity reducing strategies from domains like communication, access and monitoring, compared to less diverse states such as Vermont and West Virginia. In addition to the number, there were also differences in the approaches across states. For example, more diverse states like California utilized a ‘health equity metric’ that gauges priority for the vaccine distribution based on factors such as race, ethnicity, age, housing, transportation and education whereas less diverse states were limited to factors like age and race for vaccine prioritization. Inclusion of a multitude of factors will lead to better identification of ‘true’ vulnerable populations. Also, data monitoring and reporting in vaccine rollout plans varied across states significantly. Most states mentioned ‘monitoring’ in their plan but few had concrete descriptions for how this would be carried out. For example, Montana had limited strategies planned for reporting of race and ethnicity data in their COVID-19 vaccination reports when compared to New York. Improvements to systematic data collection and reporting can improve vaccination uptake across different strata of populations and can also inform policy makers to design and implement better targeted programs and interventions.

**Table 1:** List of inequity reducing strategies across five different domains.

Inequity Reducing Strategies by Domain	
**Prioritization:** Strategies focused to ***identify and map*** the priority population groups for vaccination.	
1	Developing a 'health equity metric' to ensure equitable delivery of vaccine. This matrix should consider race, ethnicity, income, occupation among others determinants of health.
2	Utilizing existing matrix such as CDC Social Vulnerability Index to ensure equitable delivery of vaccine till a comprehensive ‘health equity matrix’ is developed.
3	Mapping of critical populations that should be prioritized by utilizing data from hospitals, nursing and residential care facilities, law enforcement facilities, fire and rescue facilities, postal service facilities, correctional facilities
**Access:** Strategies focused to ***improve allocation and availability*** of the COVID-19 vaccination.	
1	Utilizing GIS mapping to determine distance between population and pharmacy locations in order to improve access to vaccination.
2	Providing on site vaccinations for long term care facilities.
3	Operating multiple satellite, mobile or drive‐thru clinics throughout the state in each county.
4	Opening vaccination clinics for a maximum of 12 h per day w/approx. 10 h of providing vaccinations.
5	Launching a toll-free number to provide assistance making a vaccination appointment for those could not register online.
6	Creating 'closed point dispensing sites' for emergency responders and critical infrastructure personnel not vaccinated by their employers.
7	Utilizing school-located vaccine clinics for outreach to those < 19 years and targeted community clinics for high-risk adults.
8	Expanding scope of practice for advanced EMTs and paramedics to administer vaccines.
**Communication:** Strategies focused to ***provide information*** to population regarding COVID-19 vaccine.	
1	Educating the public about the development, authorization, distribution, and execution of COVID-19 vaccines and evolving information.
2	Providing communication to all people inclusively, with respect, using non-stigmatizing, non-confusing plain bias-free language.
3	Establishing feedback mechanisms such as a web page or e-mail account to allow the audience to express concerns, ask questions, and request assistance.
4	Including toll‐free numbers or reference web pages in the message in the language of the intended audience.
5	Providing information on interpreter resources to assist with translation and sign‐language services.
6	Translating vaccination guidance, consent, and public education materials in different languages.
7	Exploring perceptions, behaviors, and message receptions to inform the effectiveness in messaging with critical and diverse communities in the state.
8	Utilizing targeting media outlets favored by the vulnerable populations/audiences.
**Safety and Trust:** Strategies focused to ***improve uptake of the vaccination*** by reducing vaccine hesitancy among the population.	
1	Ensuring public confidence in the approval or authorization process, safety, and efficacy of COVID-19 vaccines.
2	Conducting webinars on strategies to reduce vaccine hesitancy in communities of color.
**Monitoring:** Strategies focused on ***collecting and reporting data*** related to equity such as race, gender, income level etc.	
1	Ensuring real-time documentation and reporting of COVID-19 vaccine administration data from satellite, temporary, or off-site clinic settings.
2	Generating vaccination coverage level reports with various dimensions of age ranges, geography, race, ethnicity, etc.
3	Building a Vaccination Response Dashboard to monitor vaccination, including estimates of critical population categories and priority areas
4	Monitoring vaccine uptake and coverage in vulnerable and frontline populations and enhance strategies to reach populations with low vaccination uptake or coverage such as ethnic and racial minorities, people experiencing homelessness, LBGTQ+, or other groups identified as potentially vulnerable.
**Multiple Domains**	
1	Prioritizing vulnerable population groups who visit clinics whether or not they have an appointment. (Access and Prioritization)
2	Providing information to community health workers on vaccines both from a safety and efficacy perspective. (Access and Communication)
3	Notifying individuals about second dose of vaccine through phone or emails. (Access and Communication)
4	Partnering with African American churches' leader, to provide mobile vaccination sites to color, hard-to-reach communities. (Access & communication)
5	Including both paid and unpaid people serving in healthcare settings in healthcare personnel for vaccine prioritization. (Access and Prioritization)
6	Establishing points of contact and communication methods for organizations, employers, or communities within the critical population groups. (Access and Communication)
7	Collaborating and engaging with multiple partners such as pediatricians, family physicians, hospital systems, district liaisons, Tribal liaisons, Health Nursing program, state and local emergency management agencies, and community vaccinators.to encourage vaccination and provided guidance on safe vaccination during the pandemic. (Access, Safety and Trust)

We believe that these observations are informative and noteworthy. Specifically, policy makers can use the gaps identified in this investigation to strategically tailor interventions to ensure equitable access to COVID-19 vaccine. In addition, states across US can adopt and adapt different strategies identified in this investigation (Table 1) to reduce inequity in COVID-19 vaccination – both access and uptake. The summarized strategies can also be utilized as a list of options for policy makers to consider in future vaccine rollout plans. Of course, the implementation of these strategies would be time sensitive. Strategies focused on prioritization would be more important in earlier stages of vaccine rollout. For instance, identification and mapping of critical populations would be important when demand is outpacing supply of the vaccine. On the other hand, strategies focused on communication and access will be more crucial in the later phases of the vaccine rollout. For instance, partnering with religious and minority leaders, to provide vaccination to hard-to-reach communities would be important when vaccination rates are limited by vaccination hesitancy rather than supply. Future research should focus on assessing the effectiveness of these vaccine rollout strategies.

## Contributors

Anindit Chhibber: Investigation, Data curation, Formal analysis, Writing – original draft, Writing – review & editing. Aditi Kharat: Investigation, Data curation, Formal analysis, Writing – review & editing. Khanh Duong: Investigation, Data curation, Formal analysis, Writing – review & editing. Richard E. Nelson: Visualization, Writing – review & editing. Matthew H. Samore: Conceptualization, Visualization, Writing – review & editing. Fernando A. Wilson: Conceptualization, Visualization, Writing – review & editing. Nathorn Chaiyakunapruk: Conceptualization, Visualization, Writing – original draft, Writing – review & editing.

## Declaration of Interests

Khanh Duong, Richard E. Nelson, Matthew H. Samore and Nathorn Chaiyakunapruk received funds from CDC (SHEPheRD 2021 Domain 1-A015) to conduct this research. All authors reports no potential conflict of interest.
